# Traumatic intradural ruptured lumbar disc with a spinal compression fracture: A case report

**DOI:** 10.1097/MD.0000000000019037

**Published:** 2020-02-14

**Authors:** Gwangjun Lee, Moon-Soo Han, Seul-Ki Lee, Bongju Moon, Jung-Kil Lee

**Affiliations:** Department of Neurosurgery, Chonnam National University Hospital and Medical School, Gwangju, Republic of Korea.

**Keywords:** compression fracture, intradural ruptured disc, trauma

## Abstract

**Rationale:**

We present a rare case of a traumatic intradural ruptured disc associated with a mild vertebral body compression fracture along with a review of the relevant medical literature. An intradural ruptured disc often occurs due to chronic degenerative diseases and is rarely due to trauma. It can cause irreversible neurological complications if the appropriate treatment is not planned.

**Patient concerns:**

A 32-year-old male presented with motor paraparesis (grade 3/5), right ankle dorsiflexion, and great toe dorsiflexion (grade 1/5), along with radiating pain at his right L4 and L5 sensory dermatome following a fall.

**Diagnoses:**

Computed tomography revealed a compression fracture of the L2 body. Lumbar magnetic resonance imaging showed an intradural mass-like lesion on the ventral side of his spinal cord and an epidural mass-like lesion on the dorsal side of his spinal cord, indicating a hematoma.

**Interventions:**

An emergency L2 laminectomy was performed to remove the space-occupying lesions and to decompress the cauda equina and nerve root. The mass-like lesion was removed. No other lesions were found in the spinal canal.

**Outcomes:**

Pathologic examination of the intradural mass lesion revealed fibrocartilage similar to that found in disc material. The patient still continued to experience motor weakness at the 1-year follow-up examination.

**Lessons:**

We report a rare case of a traumatic lumbar disc rupture into the dural sac associated with a mild vertebral body compression fracture. Early diagnosis and prompt surgical intervention are essential, as is performing a magnetic resonance imaging or computed tomography myelogram promptly to evaluate the spinal canal when there are unexplained neurologic symptoms. An intraspinal canal evaluation should be completed before the postural reduction of the vertebral body fracture to prevent any neurological complications.

## 1 Introduction

A traumatic ruptured disc is rare, and intradural invasion is only reported in 0.26% to 0.30% of lumbar disc herniation cases.^[1]^ Trauma to the lumbar spine usually results in a bony fracture, rather than a disc rupture or paraspinal soft tissue injury. Conversely, an intradural ruptured disc typically occurs due to chronic degenerative diseases. For this reason, the ruptured disc may initially be obscured by a lumbar spine fracture and epidural hematoma.^[2–4]^ Such a lesion can cause irreversible neurological complications if an appropriate treatment plan is not made at stage of preparation for surgery. We present a case of a traumatic intradural ruptured disc that is confused at stage of diagnosis and surgical preparation and discuss possible mechanisms and clinical distinctions.

## 2 Case report

Patient has provided informed consent for publication of the case.

### 2.1 Presentation

A 32-year-old male was admitted to hospital after falling off a rock from a height of 3 m. The patient complained of back pain and lower motor weakness, in particular, right ankle dorsiflexion and great toe dorsiflexion (grade 1/5). Neurological examination revealed motor paraparesis (grade 3/5) as well as radiating pain at the right L4 and L5 sensory dermatome. There was slight leg radiating tenderness on the patient's right side with 50-degrees of knee extension. Bowel and bladder functions were normal. There were no specific problems detailed in his medical history.

### 2.2 Radiologic findings

**Figure 1:**
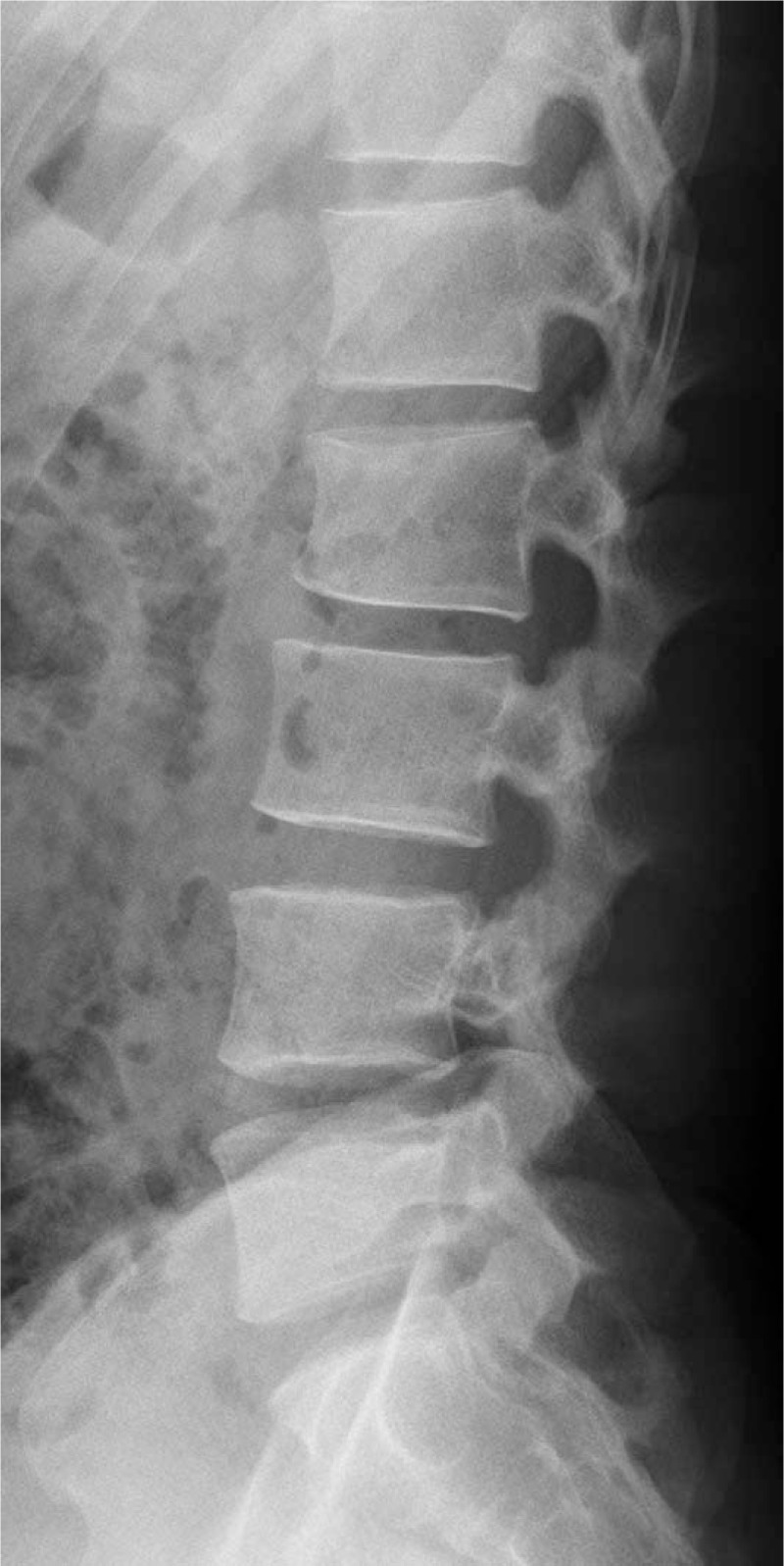
. Initial X-ray scan showing a suspected anterior compression fracture of the L2 body.

**Figure 2:**
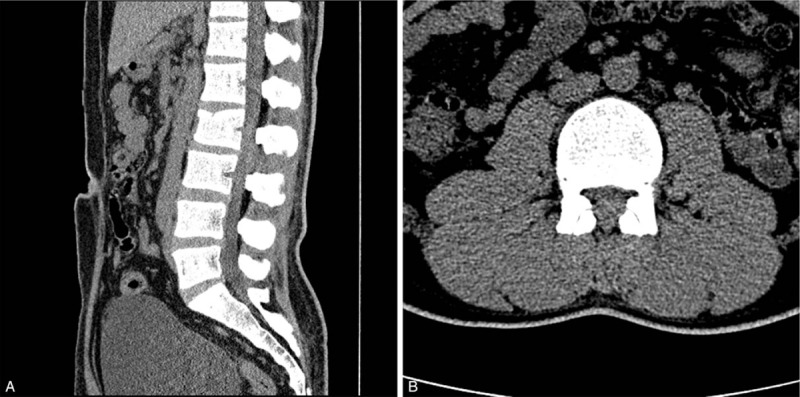
. Initial lumbar spine computed tomography scan (A and B) showing a focal compression fracture of the L2 body at the upper anterior portion and a suspected hematoma in the posterior epidural region at L2-L3.

**Figure 3:**
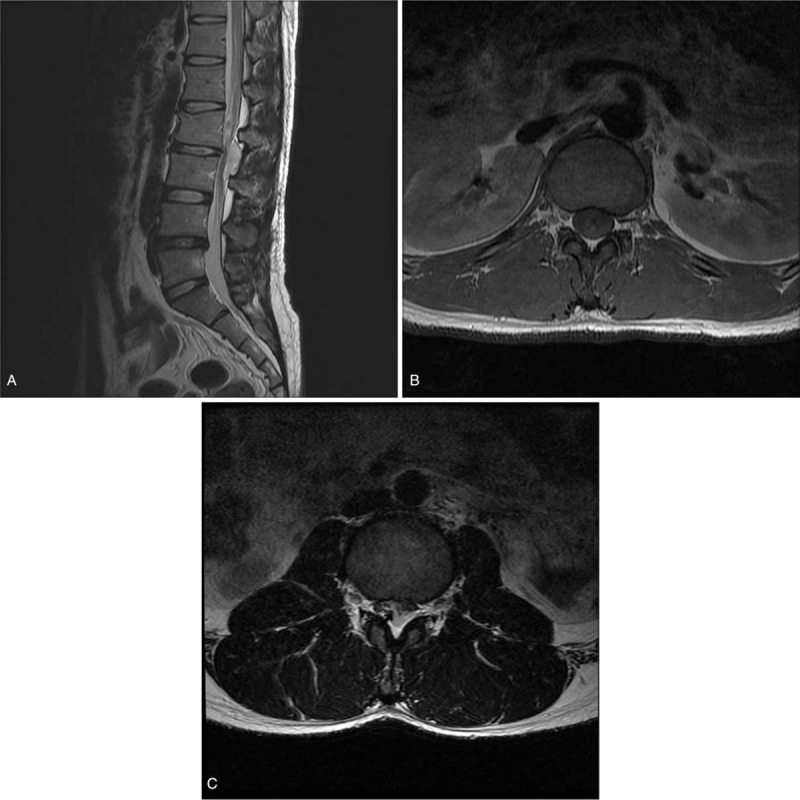
. Sagittal T2-weighted (A) MRI demonstrating a compression fracture of the L2 body at the upper anterior portion and a mass-like lesion on the intradural and posterior epidural area at L2-L3 level. Axial T1-weighted (B) and T2-weighted (C) MRI showed a deviation of the cauda equina by an intradural mass-like lesion and a lesion affecting the nerve roots and thecal sac. Two lesions (an intradural mass-like lesion on the ventral side of the spinal cord and an epidural mass-like lesion on the dorsal side) were suspected of connectivity (arrow). MRI = magnetic resonance imaging.

Lumbar plain radiographs showed a suspected compression fracture of the L2 vertebra and a slightly decreased anterior vertebral body height (Fig. 1). A lumbar spine computed tomography (CT) scan revealed a compression fracture of the L2 vertebral body at the upper anterior portion and suspected hematoma in the posterior epidural region at the L2-L3 level, but no encroachment on the spinal canal (Fig. 2Aand B). Lumbar magnetic resonance imaging (MRI) was then done to check for any neurological damage. At the L2 level, the lumbar spine MRI showed an intradural mass-like lesion on the ventral side of the patient's spinal cord. There was an epidural mass-like lesion on the dorsal side of the spinal cord at level L2-L3. The lesion showed heterogeneous iso-signal intensity in the T1-weighted image and a slightly heterogeneous high-signal intensity in the T2-weighted image as compared to the intervertebral disc (Fig. 3A–C). Two mass lesions were suspected of connectivity and nerve root compression. These lesions were believed to be a spinal hematoma, and an emergency operation was performed to improve the patient's neurological symptoms.

### 2.3 Operation procedure and findings

**Figure 4:**
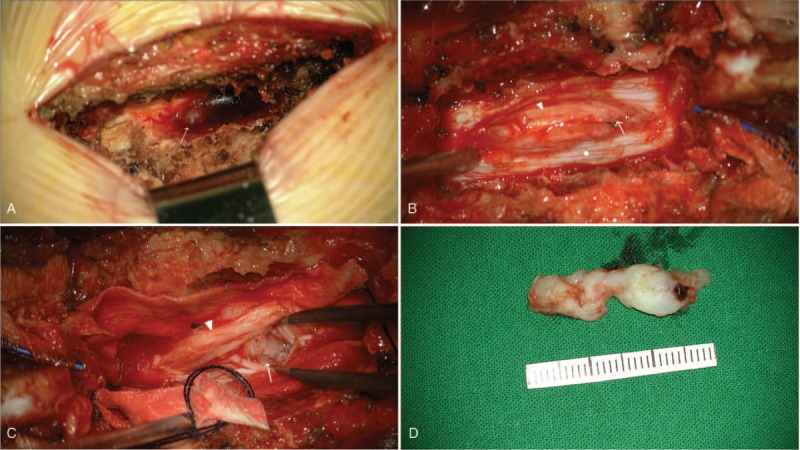
. Intraoperative photographs. After the laminectomy, penetrated material (arrow) was visible in the extradural space on the dorsal side (A). After opening the dura (asterisk), there were damaged nerve roots (arrowhead) and disc-like material visible (arrow) (B and C). The size of the lesion was 3.0 × 1.0 cm (D).

**Figure 5:**
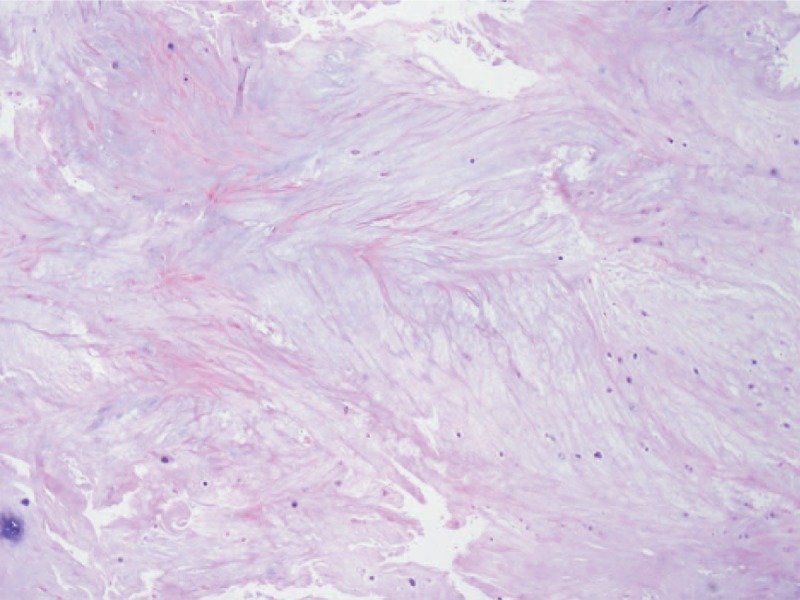
. The intradural lesion was shown to contain fibrocartilage of the intervertebral disc through a pathological examination. Some chondrocytes can be observed in this specimen, but there was no evidence of neovascularization or inflammatory response, which would indicate chronic disc degeneration.

The patient underwent a laminectomy on both sides at the L2 level. In the operative field, there was a defect on the dorsal side dura (about 3 cm) and damaged nerve roots. The ruptured disc fragment (3 cm) was exposed through the dorsal side of the dural sac from inside to outside, into the epidural space (Fig. 4A). The ruptured disc fragment was removed in 1 piece by careful microdissection (Fig. 4B–D). After removal of the mass, we explored the intradural and epidural space for other pathologic lesions. However, no other lesions were found in the spinal canal. Although we explored the anterior and ventral openings, it was challenging to identify the dural openings due to severe adhesion. As 2 sections of the vertebral column were damaged, we performed a transpedicular screw fixation for spinal stability after the dural repair on the dorsal opening using a watertight closure. To prevent cerebrospinal fluid (CSF) leakage, after the dural repair on the dorsal opening, the lateral sides of the dural sac were patched using harvested fat tissues. After confirming there was no CSF leakage using the Valsalva maneuver, Tisseel and TachoSil were applied. Pathological examination demonstrated fibrocartilage material and confirmed a disc fragment was present. (Fig. 5)

### 2.4 Postoperative course

Except lower back pain, there were no improvements in the patient's neurological symptoms immediately after surgery. The patient continued rehabilitation, and at the 1-year follow-up, their great toe motor strength had significantly improved. However, a foot drop remained on his right side. During the entire 4-year follow-up period, rehabilitation continued, and the patient's right lower extremity muscle strength improved to grade IV except for ankle dorsiflexion (grade I).

## 3 Discussion

Traumatic acute intervertebral disc herniation or rupture is common in the cervical spine.^[5–7]^ Acute traumatic disc tears and herniation are rare with lumbar spine lesions. No incidence rates appear to have been reported in the literature. Most traumatic disc ruptures in the lumbar spine accompany frequent bone injuries, such as a fracture or dislocation.^[8]^ Dandy^[9]^ first presented a case of an intradural lumbar disc rupture in 1942. The incidence is reported to be between 0.04% and 0.33% of all lumbar disc herniations.^[10]^

Ruptured disc fragments are common in the epidural area between the ventral dura and the posterior longitudinal ligament (PLL), regardless of trauma.^[3,9,10]^ In our case, a ruptured intervertebral disc invaded and penetrated the intradural space. Several mechanisms presented as the cause of the intradural disc rupture. The penetration of the PLL and dura mater is an event that precedes the intradural disc herniation.^[4]^

Dandy^[9]^ reported that pressure from the protruded disc might invade and perforate the overlying ventral dura, and Kataoka et al^[10]^ explained that tight adhesion between the ventral dura and PLL might play a role in forming an intradural disc rupture. Some authors have maintained that dura mater and dural ligaments can be found tightly adhered to the PLL and the vertebral body to the intervertebral disc in cadaveric studies.^[3,11,12]^ Causes of adhesion are congenital formation, previous operations, trauma, chronic inflammation, or osteophytes.^[13,14]^

An initial diagnosis of a traumatic intradural disc rupture using radiological imaging can be challenging due to various radiological and clinical features.^[15]^ Lesions may be obscured and underestimated on the basic radiologic examinations used for diagnosing traumatic spinal injuries, such as a simple X-ray and CT scan. Hidalgo-Ovejero et al^[16]^ reported that the existence of free air in the epidural space of a CT scan could signal an intradural ruptured disc. Since Wasserstrom et al^[17]^ made their diagnosis using a gadolinium-enhanced MRI in 1993, the rim enhancement of ruptured discs has been considered a typical MR finding for intradural ruptured discs. Choi et al^[18]^ reported signs of an intradural ruptured disc, including PLL discontinuity and a sharp beak-like appearance on an MR T2-weighted image. However, these findings may be nonspecific to an intradural lumbar disc herniation and were not observed in our case. Insufficient detail of the spinal canal can result in not diagnosing an intradural ruptured disc, resulting in a second operation being required.^[19]^

Treatment of patients with a traumatic intradural disc rupture, as well as neurological symptoms, should focus on properly removing the ruptured disc fragments. This is important as neurologic prognosis appears to be related to the preoperative duration and degree of neurologic symptoms.^[10]^

In our case study, there were several reasons for our diagnosis. The patient had no other neurological symptoms of disc herniation or spinal cord injury before the traumatic event. In the lumbar spine CT, a mild spinal compression fracture in the upper anterior portion was consistent with a disc rupture. Additionally, the lumbar MRI showed no degenerative changes in the space between the intervertebral disc spaces at the level of the compression fracture. We could exclude an intradural abscess because there were no signs or symptoms of infection, such as fever or leukocytosis.

An intradural mass was confirmed as disc material containing fibrocartilage and chondrocytes. The pathological findings revealed no evidence of chronic disc degeneration, such as neovascularization (Fig. 5). Although it was not performed in this study, immunohistochemical staining is also useful in identifying acute disc ruptures. Ito et al^[20]^ reported that negative immunohistochemical staining of the endothelial cells, macrophages, T-lymphocytes, and B-lymphocytes indicates recent nucleus pulposus extrusion.^[19]^

In many articles, dural tears on the ventral side of the spinal cord were found in the operative field. Typically, dural tears are located below the disk material. As a rule, suturing is performed to prevent CSF leakage.^[19]^ In our case, however, a ventral dural defect was not detected in the operative field either by the naked eye or microscopically. The sequestrated disc material may have migrated from the opening site of the dural perforation. The search for the dural opening on the ventral side could have caused additional damage to the spinal cord. After closing the opened dura on the dorsal side of the spinal cord at the end of the operation, CSF leakage did not occur. This may suggest that adhesions among the annulus fibrosus of disc, PLL, and dura acted as a barrier to the epidural space.

## 4 Conclusion

We describe a rare case of traumatic lumbar disc rupture into the dural sac associated with a mild vertebral body compression fracture. If a patient's neurological symptoms cannot be explained using simple radiography or a CT scan, physicians should consider soft tissue lesions, and in particular, a traumatic intradural disc rupture. An MRI or CT myelogram should then be promptly performed to evaluate the spinal canal, including the intradural space. An intraspinal canal evaluation should be performed before the postural reduction of the vertebral body fracture to prevent neurological complications occurring due to missed intradural disc fragments.

## Author contributions

**Conceptualization:** Jung-Kil Lee.

**Data curation:** Moon-Soo Han.

**Investigation:** Gwangjun Lee.

**Supervision:** Jung-Kil Lee.

**Writing – original draft:** Gwangjun Lee.

**Writing – review & editing:** Seul-Ki Lee, Bongju Moon, Jung-Kil Lee.
